# A Novel P@SiO_2_ Nano-Composite as Effective Adsorbent to Remove Methylene Blue Dye from Aqueous Media

**DOI:** 10.3390/ma16020514

**Published:** 2023-01-05

**Authors:** AbdElAziz A. Nayl, Ahmed I. Abd-Elhamid, Wael A. A. Arafa, Ismail M. Ahmed, Aref M. E. AbdEl-Rahman, Hesham M. A. Soliman, Mohamed A. Abdelgawad, Hazim M. Ali, Ashraf A. Aly, Stefan Bräse

**Affiliations:** 1 Department of Chemistry, College of Science, Jouf University, Sakaka 72341, Aljouf, Saudi Arabia; 2 Composites and Nanostructured Materials Research Department, Advanced Technology and New Materials Research Institute, City of Scientific Research and Technological Applications (SRTA-City), New Borg Al-Arab 21934, Egypt; 3 Department of Pharmaceutical Chemistry, College of Pharmacy, Jouf University, Sakaka 72341, Aljouf, Saudi Arabia; 4 Chemistry Department, Faculty of Science, Organic Division, Minia University, El-Minia 61519, Egypt; 5 Institute of Organic Chemistry (IOC), Karlsruhe Institute of Technology (KIT), Fritz-Haber-Weg 6, 76133 Karlsruhe, Germany; 6 Institute of Biological and Chemical Systems-Functional Molecular Systems (IBCS-FMS), Hermann-von-Helmholtz-Platz 1, 76344 Eggenstein-Leopoldshafen, Germany

**Keywords:** nanocomposite, adsorption, eco-friendly, methylene blue, wastewater

## Abstract

This work aims to prepare a novel phosphate-embedded silica nanoparticles (P@SiO_2_) nanocomposite as an effective adsorbent through a hydrothermal route. Firstly, a mixed solution of sodium silicate and sodium phosphate was passed through a strong acidic resin to convert it into hydrogen form. After that, the resultant solution was hydrothermally treated to yield P@SiO_2_ nanocomposite. Using kinetic studies, methylene blue (MB) dye was selected to study the removal behavior of the P@SiO_2_ nanocomposite. The obtained composite was characterized using several advanced techniques. The experimental results showed rapid kinetic adsorption where the equilibrium was reached within 100 s, and the pseudo-second-order fitted well with experimental data. Moreover, according to Langmuir, one gram of P@SiO_2_ nanocomposite can remove 76.92 mg of the methylene blue dye. The thermodynamic studies showed that the adsorption process was spontaneous, exothermic, and ordered at the solid/solution interface. Finally, the results indicated that the presence of NaCl did not impact the adsorption behavior of MB dye. Due to the significant efficiency and promising properties of the prepared P@SiO_2_ nanocomposite, it could be used as an effective adsorbent material to remove various cationic forms of pollutants from aqueous solutions in future works.

## 1. Introduction

Many hazardous materials, such as heavy metals, dyes, drugs, pesticides, etc., have been discharged into the aquatic environment. This water pollution has become a severe universal subject and attracts attention worldwide from researchers, politicians, and simple people. Dyes are one of the more industrial effluents and are heavily used in several industries, such as food, wood, leather, paper, silk, etc. Discharging dyes in the aquatic sphere, even at low concentrations, will harm all living organisms that live in water, animals, and humans, where it is a toxic, carcinogenic, mutagenic, and non-degradable materials that can stay in the environment for a long time [1]. Methylene blue (MB) dye is the most famous water contaminant that badly impacts health through abdominal disorders, respiratory distress, skin sensitization, and blindness [2,3,4]. Also, methylene blue, with its deep blue color, reduces the penetration of the light for aquatic organisms, which has a bad effect on the environment and disorders the balance of the ecosystem leading to dangerous issues for all forms of living systems and thus threatens their life [1,5,6,7]. Therefore, it is very important to decontaminate this dye from water to prevent its discharge into the environment, especially aquatic bio-systems [1]. So, the remediation of such pollutants has extraordinary value due to the water shortage that many countries face.

Several strategies have been investigated and developed to purify water from such harmful material, including photocatalytic degradation, solvent extraction, coagulation, biodegradation, oxidation, zonation, and adsorption [5,8,9]. Adsorption, among the techniques, is the most powerful, economical, and efficient technique utilized to decontaminate dyes from wastewater at low concentrations due to its simplicity, low cost, and not requiring advanced technology [10,11,12,13]. During this year, novel adsorbent materials have been reported to remove toxic dyes such as methylene blue from wastewater [14,15,16,17,18,19,20,21,22,23,24,25,26,27]. Activated carbon (AC) is one of the most traditional, effective, and extensively utilized adsorbents in adsorption technologies, and it is among the cheapest sorbents in market. However, its failure to regenerate is still considered a major setback [28], and recycling its powdered form from the liquid phase is another intractable issue [29]. Also, some investigated materials’ main shortcomings cause them to be considered undesirable adsorbent materials due to their relatively higher cost and generation of secondary contaminants. Therefore, selecting proper adsorbent materials that produce low secondary contaminants with favorable chemical and thermal stability as well as suitable regeneration operations is the key to adsorption technologies [13]. So, scientists direct their works to investigate other alternative natural materials to treat the colored dyestuff effluents to overcome such disadvantages [30]. Siliceous adsorbents, such as silica, perlite, and glass fibers, are cost-effective and found naturally with good biocompatibility, excellent performance with non-toxicity, and considerable thermal stability in various applications, such as separation [31]. Due to the lower toxicity, easy availability, eco-friendly synthesis processes, cost-effectiveness, and bioactivity of SiO_2_-NPs, it is safely used in food industries, drug delivery, pharmaceutical, and water treatment systems [9,32]. Also, it has higher efficiency in removing dyes such as MB dye, where silica nanoparticle adsorbents are characterized by high surface area, low toxicity, high stability, and economical preparation that enable them to serve as an efficient adsorbent in the water treatment processes. To enhance the removal capacities of such silica nanoparticles, the surface of the material has been modified by other materials [32]. Therefore, several processes were applied to surface silica particles for water treatments [33], especially in dye removal. The first is a reduction in particle size to nanoscale to obtain a great specific surface area [33]. Surface silica nanoparticles are also hybridized, functionalized, magnetized, and doped with polymers [33,34]. One or more of these processes is carried out to increase the adsorption capacity [34]. The presence of surface (-OH) groups attached to the Si-atom on SiO_2_NPs is a very important characteristic and are termed silanols [34]. These silanols can interact selectively with dyes and are improved by changing the pH values [34]. Recently, many works have reported a great achievement for the new generation of silica-based nanomaterials, which illustrates outstanding adsorption capacities for several dyes by synthesis, functionalized, and hybrid SiO_2_NPs [34]. Modifying the silica nanoparticles’ surface will improve the adsorption performance [35]. Also reported in the literature, the surface of the silica nanoparticles was modified with organic chains involved in silane compounds. Jesionowski and Krysztafkiewicz [36] precipitated silica nanoparticles in an acidic solution followed by coupling with hydrophobic/hydrophilic function groups. Lee and Jo **[37]** prepared silica nanoparticles using the Stöber method and functionalized them with methyltriethoxysilane. Hah and Koo **[38]** synthesized silica nanoparticles using tetraethyl orthosilicate (TEOS) under a basic environment, and then methyltrimethoxysilane (MTMS) and vinyltrimethoxysilane (VTMS) were employed for anchoring the surface of the nanoparticles.

In previous studies, the modification of the silica nanoparticles was prepared through discontinuous processes with different steps and required expensive chemicals. Therefore, in our present study, tri-sodium phosphate (Na_3_PO_4_), an inexpensive and valuable material, was grafted into silica nanoparticles through a simple and green hydrothermal route. Trisodium phosphate and sodium silicate were dissolved in an aqueous solution, passed through highly acidic resin, and finally, hydrothermally treated to produce a white precipitate (P@SiO_2_ nanocomposite) which will be used as an effective adsorbent to the methylene blue (MB). The characterization of the fabricated P@SiO_2_ nanocomposite was obtained with various physicochemical techniques such as scanning electron microscopy (SEM), X-ray diffraction analysis (XRD), elemental dispersive energy (EDX), and Fourier-transform infrared spectroscopy (FTIR). Also, the influences of the adsorption conditions, including P@SiO_2_ nanocomposite dosage, initial MB dye concentrations, and solution pH, on the removal efficiencies were investigated. Also, the thermodynamic, kinetic and regeneration properties were studied.

## 2. Materials and Methods

### 2.1. Materials

Di-sodium silicate (Na_2_SiO_3_, Sigma-Aldrich, St. Louis, MO, USA), tri-sodium phosphate (Na_3_PO_4_·12H_2_O, 96%, Sigma-Aldrich), strong acid type of cation exchange resin (Rohm and Haas, Valbonne, France) and methylene blue (Sigma-Aldrich), NaCl (Sigma-Aldrich) were used.

### 2.2. Characterization and Analysis

The surface morphologies of the nanocomposite were detected using a scanning electron microscope (SEM, JEOL GSM-6610LV. Japan) operating at an acceleration voltage of 20 kV. The surfaces of the specimen were treated with a thin layer of gold before imaging. The dimension of the nanomaterial was measured using Image J software (Copyright 1998-2003 JEOL LDT) from the SEM captures in an original magnification of 30,000× and 50,000×. At least 25 isolated nanoparticles were randomly selected, and their diameters and diameter distributions were measured and averaged. The elemental analysis of the materials before and after the adsorption process was determined by the EDS unit connected with the SEM. The infrared spectrum of the nanoparticles was investigated by Fourier transform infrared spectrometer (FTIR, Shimadzu FTIR-8400 S, Japan) FTIR spectra. The infrared spectra were recorded in the transmission mode using nanomaterials mixed with KBr. The experiments were investigated in the range of 4000–400 cm^−1^. The crystal structure of the composite was described by X-ray diffraction (XRD, Shimadzu, Japan XRD-7000) with a scanning speed of 12° min^−1^ from 5 to 100°.

### 2.3. Preparation of P@SiO_2_ Nanocomposite

In a typical experiment, 9.0 g of di-sodium silicate and 2.0 g of tri-sodium phosphate were dissolved in 50 mL of double distilled water. After that, the previously prepared solution was loaded on a strong acid type of cation exchange resin column. The acidified solution was recovered from the column by elution. The eluted solution was charged into stainless steel autoclave reactor and placed in a muffle at 150 °C for 24 h. The resulting white powder centrifuge was washed several times with double distilled water and dried at 70 °C for 24 h. Figure S1 (in Supplementary Materials) shows the schematic diagram of the prepared powder.

### 2.4. Adsorption Studies

A methylene blue (MB) dye stock solution was investigated by stirring 1.0 g of the solid dye in 1.0 L of double distilled water, and the required concentrations were obtained by dilution. Batch removal experiments were prepared by stirring 10–25 mg of nanopowder (NPs) with 10 mL aqueous solution of methylene blue in 50 mL flasks at different concentrations (100–300 mg/L), pH (1.5–11), temperatures (25–80 °C), sodium chloride dose (0–2 g) and adsorbent tests take place at constant stirring speed. The nanopowder was isolated from the MB dye solution by centrifugation. The dye concentration residue was analyzed using UV–vis spectroscopy at λ = 664 nm. The dye removal percent, %R, can be measured by applying the Equation (1):

(1) $$\%R=\frac{C_{o}-C_{e}}{C_{o}}\times 100$$

where Cₒ and C_e_ are the initial and equilibrium concentrations of the liquid phase of the dye (mg/L), respectively.

### 2.5. Mathematical Modeling

Adsorption kinetic, isotherm, and thermodynamic models investigated in our study are explained in the Supplementary Material file (Sections S1.1–S1.3, respectively).

## 3. Results and Discussion

### 3.1. Characterizations

**Figure 1:**
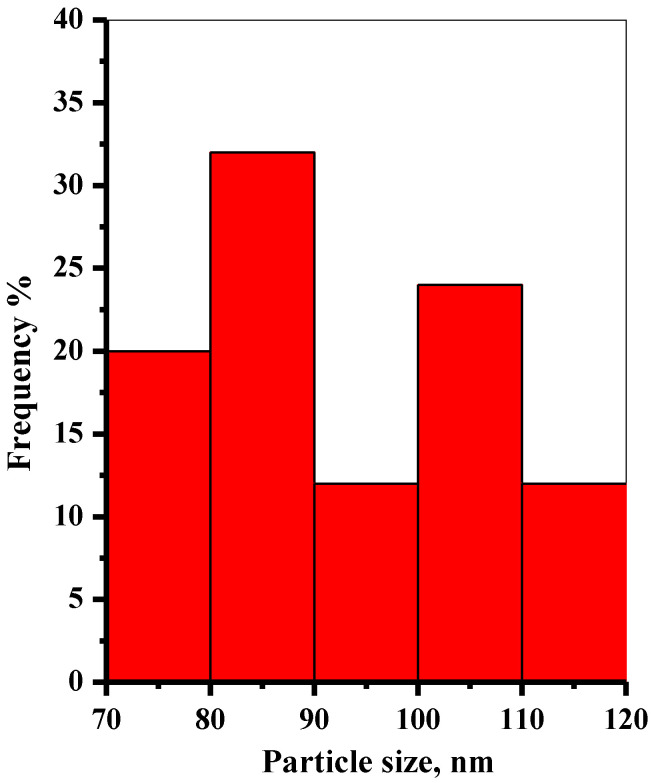
Histogram for the particle size distributions of the prepared P@SiO_2_-nanoparticles.

Scanning electron microscopy (SEM) was applied to study the surface morphologies of the prepared P@SiO_2_ nanocomposite. The SEM images of typical phosphate-doped silica (Si-P) nanoparticles are shown in Figure S1a–d. The prepared nanoparticles are spherical and have a diameter range (85–173 nm). The dimensions of the P@SiO_2_ nanoparticles were determined using image J software from the SEM captures in an original magnification of 30,000× and 50,000×. The average diameters and diameter distributions for 25 randomly selected isolated nanoparticles were measured and averaged, as represented in Figure 1. The data obtained showed that the morphologies of the synthesized P@SiO_2_ nanocomposite are relatively homogenous.

**Figure 2:**
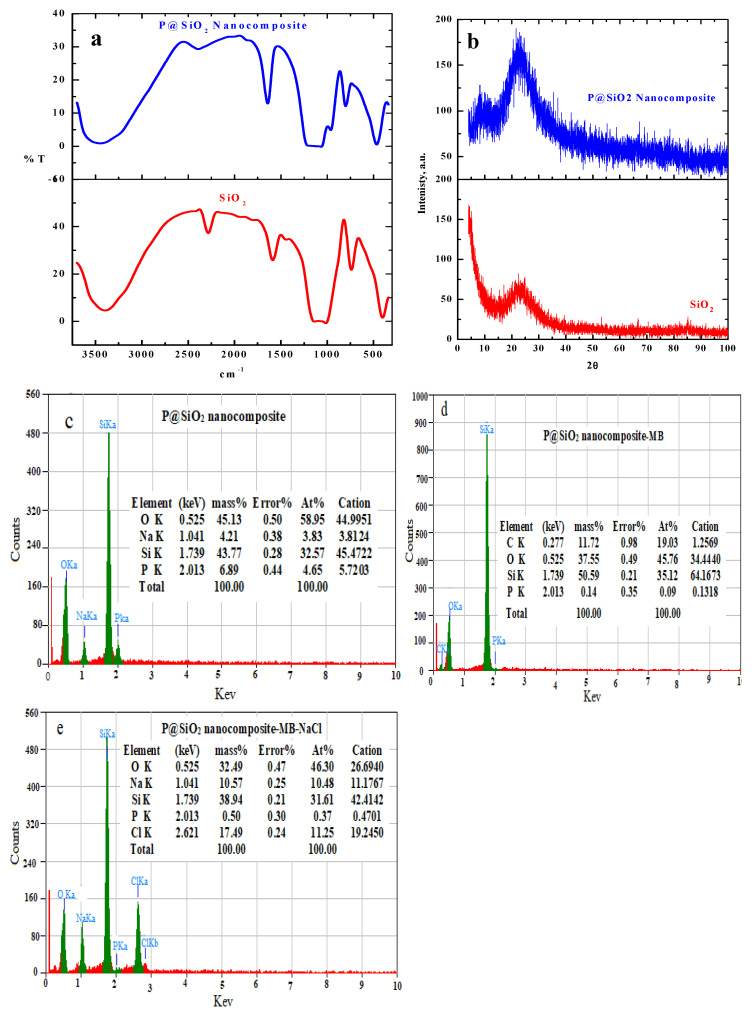
(**a**) FTIR spectrum of SiO_2_ and P@SiO_2_ nanoparticles, (**b**) XRD pattern of SiO_2_ and P@SiO_2_ nanoparticles, and EDS analysis of (**c**) Si-P and (**d**) P@SiO_2_-MB and (**e**) P@SiO_2_-MB-NaCl powder.

FTIR spectra of silica and P@SiO_2_ nanocomposite were investigated to obtain the basic information that illustrates the chemical structures of the prepared adsorbent material, as shown in Figure 2a.

The common bands assigned to various vibrations of SiO_2_ were observed. A broad band centered at around 3401 cm^−^^1^ corresponds to the stretching bands of the H-bonded H_2_O molecules in the interlayer [35]. The adsorbed water molecules show a bending vibrations band at 1597 cm^−^^1^ [35]. Two strong bands appear at 1016 cm^−^^1^ and 1136 cm^−^^1^ corresponding to the Si-O-Si asymmetric stretching vibrations [33]. Furthermore, the symmetric stretching and the bending mode vibrations of Si-O-Si appear at 738 cm^−^^1^ [39] and 405 cm^−^^1^, respectively. Upon addition of phosphate to form Si-P, these peaks have a shift (405→467, 738→800, 1016→1066, 1136→1226, 1597→1646, and 3401→3465) [40]. A strong band at 1066 and 956 cm^–1^, a characteristic of a PO_4_^−^^3^ group, was detected [41]. All of these mentioned spectral data prove that the P@SiO_2_ nanocomposite was successfully prepared and has many active groups on the prepared P@SiO_2_ nanocomposite surface that enhance the adsorption processes.

X-ray diffraction patterns were investigated to obtain information about the internal structures of SiO_2_ and P@SiO_2_ nanocomposites. The data obtained showed a broad peak at 2θ ≈ 20° and broad peaks due to the amorphous nature of the synthesized P@SiO_2_ nanocomposite, as represented in Figure 2b [42]. Also, other diffraction peaks were not observed at 2θ = 0.5–10°, due to the exchanges of hydrated protons and cations between the interlayers [35].

EDS measurements of P@SiO_2_ nanocomposite (Figure 2c)**,** P@SiO_2_-MB (Figure 2d), and P@SiO_2_-MB (Figure 2e) in the presence of NaCl (P@SiO_2_-MB-NaCl) are presented in Figure 2c. The diagram shows that the prepared P@SiO_2_ powder consists of Si, O, P, and Na, as demonstrated in Figure 2c. After interaction with the MB dye, the introduction of C is detected in the analyzed powder (P@SiO_2_-MB) (as illustrated in Figure 2c). Moreover, to evaluate the influence of ionic strength on the adsorption of MB dye onto P@SiO_2_ nanocomposite, the elements of Na and Cl were observed in the powder (P@SiO_2_-MB-NaCl). This result proves that NaCl does not has any influence on the adsorption process under the investigated conditions

### 3.2. Adsorption Study

#### 3.2.1. Effect of Contact Time and P@SiO_2_ Nanocomposite Dose

**Figure 3:**
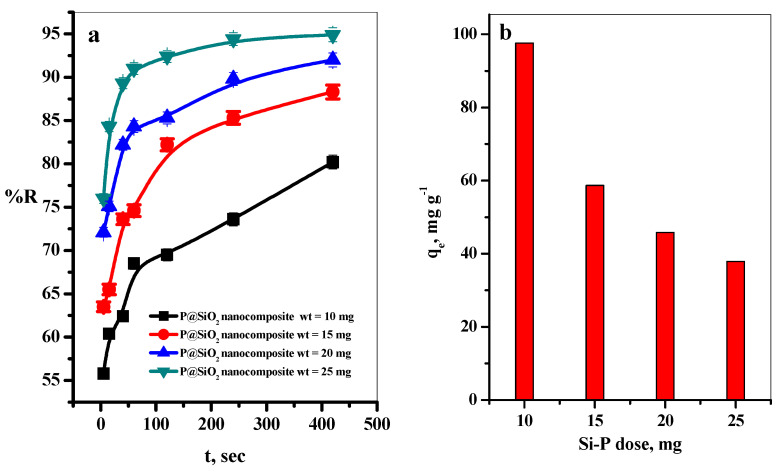

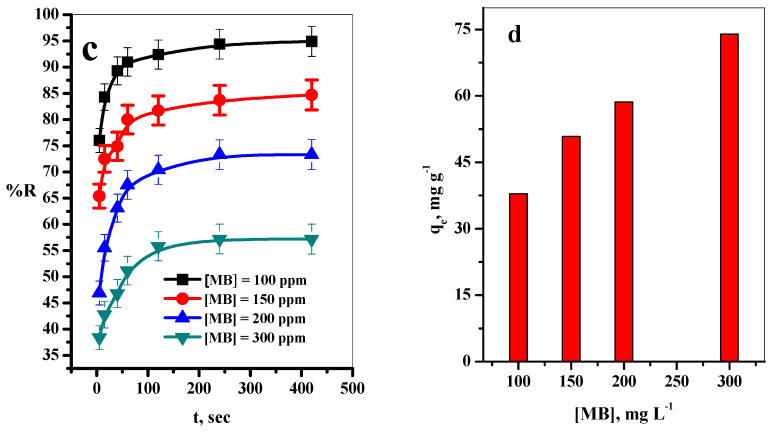
Effect of (**a**) P@SiO_2_ nanocomposite doses at the different contact times, (**b**) effect of P@SiO_2_ nanocomposite doses on q_e_ values, (**c**) effect of initial MB concentrations at the different contact time, and (**d**) its effect on q_e_ values, on the adsorption of MB dye. (P@SiO_2_ dose ([MB] = 100 mg/L/10 mL, pH = 7, T = 25 °C) and (**d**) initial MB concentrations ([P@SiO_2_] = 25 mg/10 mL, pH = 7, T = 25 °C).

The effect of contact time on the adsorption percentage (%R) of MB dye was tested using various P@SiO_2_ nanocomposite adsorbents mixed with a defined concentration of MB dye (10–25 mg/10 mL) at a period of 0.0–400 s. The removal percentages (%R) of the MB dye increased linearly with the time increase to 100 s (Figure 3a). This is an attractive property of the promising P@SiO_2_ nanocomposite and gives it added value to use as an effective and economical adsorbent material in wastewater treatment processes.

To study the effect of the P@SiO_2_ nanocomposite dose on the uptake of the MB dye species, various weights of P@SiO_2_ (10–25 mg/10 mL) were tested at constant other conditions ([MB] = 100 ppm, pH = 7, and T = 25 °C), as shown in Figure 3b. Also, it can be observed that the (%R) increased from 80.2 to 94.8%, with a further increase in the adsorbent dose from 10 to 25 mg, as illustrated in Figure 3b. This is attributed to increasing the number of active sites with an increase in the adsorbent dose, which enhances the removal percentage of MB dye molecules [43]. On the other hand, the increase in the adsorbent dose leads to a reduction in the amount of dye adsorbed to one gram of the adsorbent; this causes a decrease in q_e_ value as the adsorbent dose increase [43], as shown in Figure 3b. Figure S2 represented the relation between q_e_ vs. C_e_ at different (a) P@SiO_2_ nanocomposite doses and (b) initial MB concentrations.

#### 3.2.2. Effect of Initial MB Dye Concentration

The effect of the initial MB dye concentrations on the removal percent (%R) and the equilibrium adsorption amount of the MB dye by P@SiO_2_ nanocomposite from an aqueous solution was investigated (as in Figure 3c) using initial MB dye concentrations ranging from 100 to 300 mg L^−^^1^ at adsorbent dose = 25 mg/10 mL, pH = 7, and T = 25 °C. It was observed that the %R increased with the time of the initial concentration to 100 s. After that, the equilibrium state was reached, as shown in Figure 2c. Moreover, the adsorption capacity of MB dye (q_e_) increases with a further increase in the initial dye concentration in the range (100–300 mg L^−^^1^), as presented in Figure 3d. The reason for this may refer to the increase in the MB dye concentrations causing improvement in the concentration gradient’s driving force, which accelerates the MB dye species’ diffusion velocity into the P@SiO_2_ nanocomposite adsorbent particles [44]. Then, the concentration gradient reduces due to the adsorption of MB molecules on the active sites of the P@SiO_2_ nanocomposite.

In other words, at higher initial concentrations of MB, the binding sites of P@SiO_2_ nanocomposite adsorbent were encompassed with many MB species in the solution. Hence, the adsorption capacity of the P@SiO_2_ nanocomposite was enhanced by increasing the MB concentration, enhancing the adsorption capacity [45].

#### 3.2.3. Effect of pH

**Figure 4:**
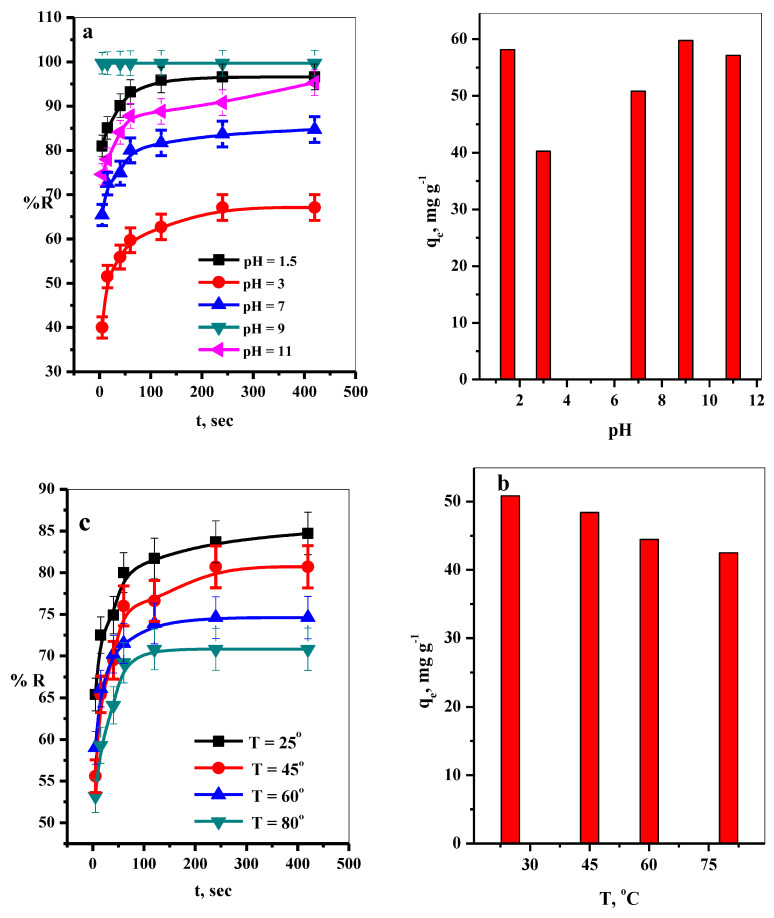
Effect of (**a**) pH, (**b**) effect of pH on q_e_ values, (**c**) effect of temperature, and (**d**) its effect on q_e_ values, on the adsorption of MB dye at different contact times ([P@SiO_2_] = 25 mg, [MB] = 150 mg/L/10 mL, T = 25 °C) and (**d**) temperature ([P@SiO_2_] = 25 mg, [MB] = 150 mg/L/10 mL, pH = 7).

The pH of the aqueous media is a very important factor in the adsorption process. The pH value affects the surface charge of the P@SiO_2_ nanocomposite adsorbent and the degree of the ionization of the MB dye species. The effect of the pH was studied in the range 1.5–11 (by adjusting the pH with HCl (0.1 N) and NaOH (0.1 N) solutions) ([MB dye] = 150 ppm, [P@SiO_2_ nanocomposite] = 25 mg/10 mL, T = 25 °C) on the removal percent %R of the MB dye by P@SiO_2_ nanocomposite was performed in Figure 4a. It was observed that the removal percentages were changed with further variation in the pH values as the following; at pH = 1.5, 3.0, 7.0, 9.0, and 11.0 the %R was 96.6, 67.1, 84.7, 99.7, and 95.3%, respectively. Consequently, the maximum adsorption capacities varied according to the pH values, as shown in Figure 4b.

Therefore, with an increase in the pH value of 3–9, the active groups on the P@SiO_2_ nanocomposite are deprotonated to carry negative charges, leading to electrostatic interactions with MB molecules increasing the removal percentages. At pH > 9, the removal percentage decreased, and this may be attributed to the –OH-anions of NaOH which can inhibit the electrostatic interactions between P@SiO2 nanocomposite and MB molecules by blocking the positive charges on MB molecules surfaces [46].

The color intensity of the MB dye sample varied according to pH value and is represented in Figure S3.

#### 3.2.4. Effect of Temperature

The influence of the temperature, ranged from 25 to 80 °C, on the %R of the MB dye from aqueous media onto P@SiO_2_ nanocomposite at pH = 7, [MB] = 150 ppm, [P@SiO_2_ nanocomposite] = 25 mg/10 mL was investigated and is represented in Figure 4c. It was found that the %R of the MB dye increased with the time the temperature degrees were used overall. Consequently, the removal percentages decreased as the temperature increased, as illustrated in Figure 4c. Furthermore, as the dye solution temperature rose, the adsorbate’s maximum adsorption capacity decreased [47], as plotted in Figure 4d. This can be attributed to the exothermic nature of the adsorption process.

#### 3.2.5. Adsorption Kinetics

**Figure 5:**
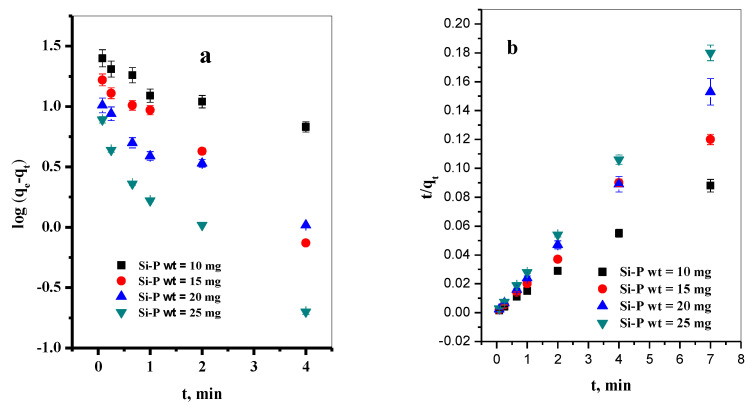

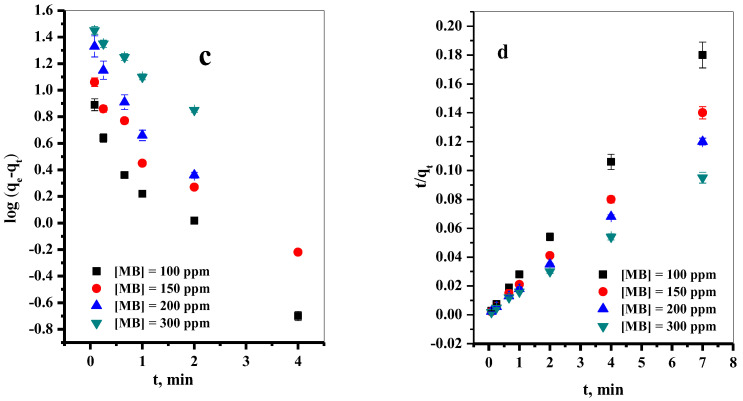
(**a**) Effect of contact time on removal percent of MB dye, (**b**) P@SiO_2_ nanocomposite dose on equilibrium constant, (**c**) pseudo-first-order plot, (**d**) pseudo-second-order plot. ([P@SiO_2_] = 10–25 mg/10 mL, [MB] = 100 ppm, pH = 7, T = 25 °C).

The kinetics of the MB dye adsorption on the P@SiO_2_ nanocomposite for various P@SiO_2_ nanocomposite doses were tested in contact times ranging from 0.0 to 7.0 min, and the results obtained are illustrated in Figure 5a,b. The calculated kinetic parameters are summarized in Table 1. As shown in Table 1, the correlation coefficient (R^2^) related to the pseudo-second-order is higher than that obtained for the pseudo-first-order. Moreover, the calculated maximum adsorption capacity for the pseudo-second-order matches well with the experimental data. This indicated that the adsorption kinetics of various P@SiO_2_ nanocomposite doses were described very well with the pseudo-second-order. For this model, it is suggested that the rate-limiting step might be chemical adsorption for the adsorption of MB dye onto P@SiO_2_ nanocomposite [23].

The mechanism and kinetics of the removal of MB dye onto P@SiO_2_ nanocomposite nanoparticles were evaluated by applying the data obtained from the dye concentration experiment using the pseudo-first-order (Equation (S3)) and pseudo-second-order models (Equation (S5)). Moreover, the kinetic parameters obtained from the linear plots (Figure 5c,d) of the two models were calculated and recorded in Table 2. Referring to the values of R^2^, the experimental data of the adsorption of MB dye on the P@SiO_2_ nanocomposite showed a better fit with the pseudo-second-order models, which indicates that the dye species was chemically adsorbed and the adsorbent surface is the rate-limiting step [48].

**Figure 6:**
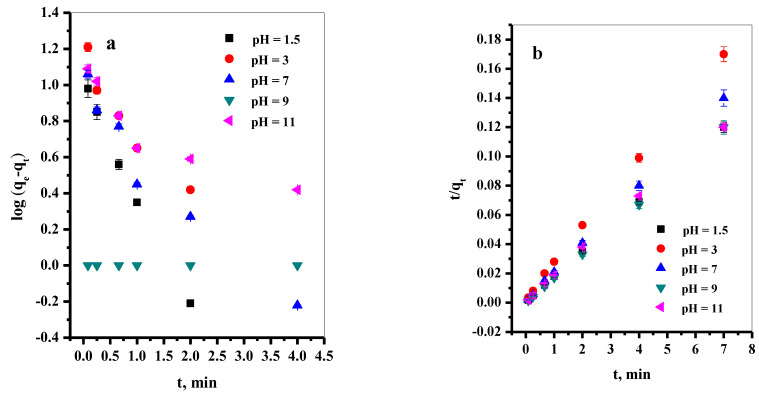

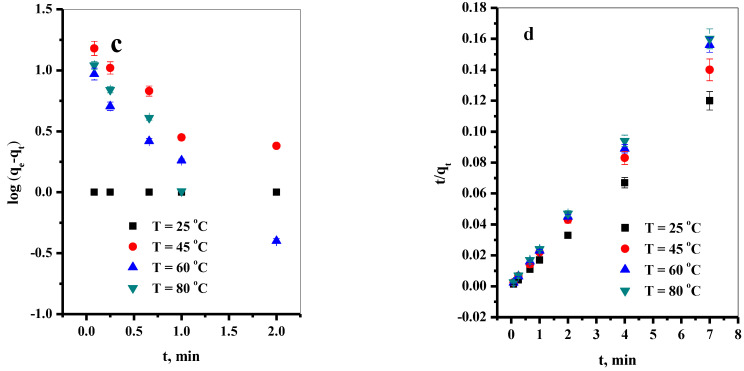
(**a**) Effect of contact time on removal percent of MB dye, (**b**) pH on the equilibrium constant, (**c**) pseudo-first-order plot, (**d**) pseudo-second-order plot (pH = 1.5–11, [MB] = 150 ppm, [P@SiO_2_] = 25 mg/10 mL, T = 25 °C).

By plotting log (q_e_–q_t_) versus t at different pH values of the MB dye solution (Figure 6a,b), the correlation coefficient (R^2^), the first-order rate constant (k_1_), and q_e_ were calculated from the slopes and intercepts of the straight lines and listed in Table 3. Similarly, R^2^, k_2_, and q_e_ related to the pseudo-second-order were calculated from the linear plot of the t/q_t_ versus t at different pH values of the dye solution and recorded in Table 3. By comparing the values of R^2^ related to the pseudo-first-order kinetic model with that in the case of the pseudo-second-order kinetic model, it can be observed that the R^2^ in the pseudo-second-order kinetic model is higher than in the pseudo-first-order kinetic model. Moreover, the calculated q_e_ value obtained from the pseudo-second-order kinetic model is closer to the experimental q_e_ values. This indicates that the pseudo-second-order kinetic model best describes the adsorption kinetics rather than the pseudo-first-order model. To investigate the mechanism of the adsorption of MB dye at various temperatures, the Lagergren pseudo-first-order kinetic and pseudo-second-order kinetic models were applied (Figure 6c,d). The revealing parameters of the two models were evaluated and summarized in Table 4. According to the data obtained, it can be decided that the pseudo-second-order equation is the better-fitting model. This is due to it owning higher R^2^ values [48]. In addition, the calculated maximum adsorption capacities, q_e_, from the pseudo-second-order model are close to the values of the experimental ones. This demonstrated that surface control mainly explains the adsorption processes rather than adsorbate diffusion. These results illustrated that chemical bonding or chemisorption between MB dye molecules and the active sites on the surface of P@SiO_2_ nanocomposite might dominate the adsorption process, and this result agrees with what was reported in the literature [29].

#### 3.2.6. Adsorption Isotherm

The most famous isotherms used to describe the adsorption isotherm are Langmuir, Freundlich, and Tempkin isotherm expressions given by Equations (S6)–(S8), respectively.

**Figure 7:**
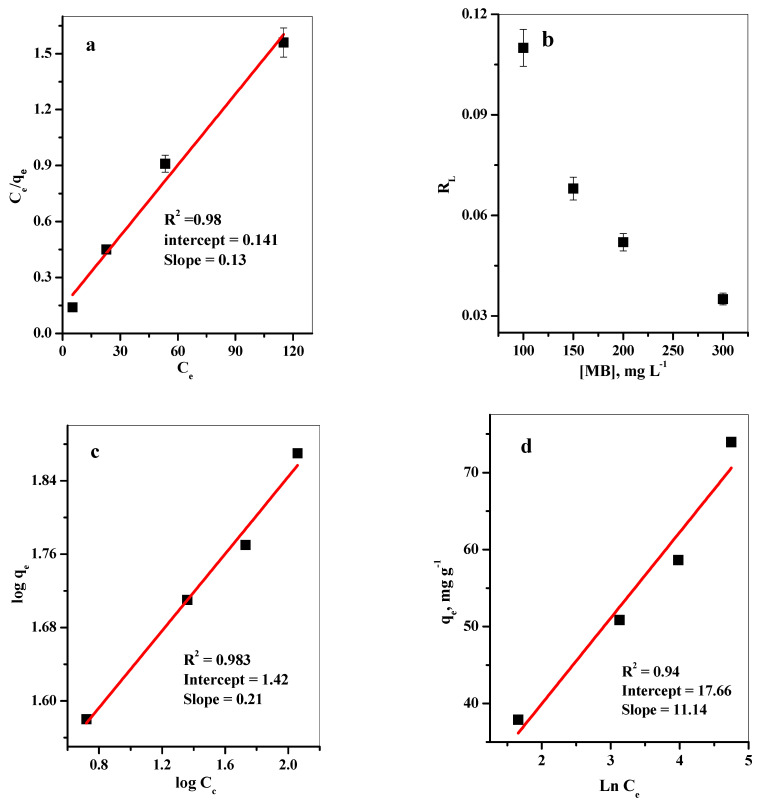
Adsorption isotherms: (**a**) Langmuir isotherm plot (**b**) Freundlich isotherm plot (**c**) R_L_ (**d**) Tempkin isotherm plot for removal of MB dye (t = 42. s, ([MB] = 100–300 ppm, [dose] = 25 mg/10 mL, pH = 7, T = 25 °C).

Langmuir isotherm supposes a monolayer of the adsorbate adsorbed on homogenously active sites with the same adsorption energies. Moreover, once these sites are occupied, no more adsorption takes place. Langmuir constants Q° and b (Table 5) can be obtained from the slope and intercept of the linear plot of C_e_/q_e_ versus C_e_ as illustrated in Figure 7a.

Dimensionless separation factor (R_L_) for the MB dye adsorption onto the P@SiO_2_ nanocomposite surface was determined from Equation (S6). If R_L_ > 1, unfavorable; R_L_ = 1, linear; 0 < R_L_ < 1, favorable; R_L_ = 0, irreversible (Figure 7b). It can be observed that the R_L_ values between 0 and 1 indicate a favorable adsorption process.

The Freundlich isotherm suggested a heterogeneous surface with non-equivalent energetic binding sites. The Freundlich constants can be calculated by plotting ln q_e_ versus ln C_e_ (Figure 7c and Table 5). From the data in Table 5, 1/n < 1, which suggests a normal Langmuir isotherm.

The adsorption performance of MB dye onto P@SiO_2_ nanocomposite was assessed by referring to the Tempkin isotherm model (Equation (S8)), and the linear relationship is plotted in Figure 7d. The correlation coefficient (R^2^ = 0.940) shows the poorest fit to the experimental adsorption equilibrium data, as summarized in Table 5.

The obtained values of the correlation coefficient (R^2^) (≈0.98) suggesting that the adsorption isotherm data of the adsorption of MB species onto P@SiO_2_ nanocomposite fits better for both Langmuir and Freundlich isotherm models. Based on the closest values of the experimental and calculated Q_max_, the adsorption results of MB species onto P@SiO_2_ nanocomposite were fitted well with the Langmuir model representing monolayer adsorption on homogeneous surfaces [17,26]. Hence, the MB species sequestration processes occurred at P@SiO_2_ nanocomposite surfaces via the monolayer adsorption systems [26,47].

#### 3.2.7. Adsorption Thermodynamics

Thermodynamic parameters were determined from the linear plot of Ln K_c_ vis T^−^^1^ (Figure S4) and according to Equations (S8)–(S10) and summarized in Table 6. The negative ∆G° values make it clear that the adsorption of MB dye on P@SiO_2_ nanocomposite is a spontaneous adsorption process. Moreover, the decrease showed for the ∆G° values with the increasing temperature from 298 to 353 K, demonstrating the adsorption performance is favored at lower temperatures and the adsorption of MB species onto P@SiO_2_ nanocomposite is a spontaneous process [26,35]. The ∆H° had a negative value confirming the exothermic nature of the adsorption process. The negative value of ∆S° suggests decreasing in the randomness of the solid/solution interfaces [26]. Activation energies, Ea, lower than 42 kJ/mol, suggest a diffusion-controlled mechanism, and higher than that value exhibits chemisorptions behavior. Here, the calculated value of E_a_ is 45.3 kJ mol^−^^1^; this indicates that the adsorption of MB onto P@SiO_2_ nanocomposite is a chemically controlled process. The data represented in Figure 4b confirms this result. Also, the P@SiO_2_ nanocomposite illustrates excellent efficiency in adsorbing MB dye molecules from both acidic and alkaline media. Therefore, we can conclude that the P@SiO_2_ nanocomposite will not be regenerated well. This could be considered an advantage where the MB dye molecules will be restricted from release again into the surrounding environment after adsorption due to the chemical bonding or chemisorption between MB dye molecules and the active groups on the surface of the P@SiO_2_ nanocomposite.

#### 3.2.8. Effect of Ionic Strength on the Adsorption Percent of MB Dye

In practical application, studying the effect of NaCl dose is very important to evaluate the influence of ionic strength on the adsorption percent of MB dye onto P@SiO_2_ nanocomposite. Here, we study the effect of NaCl dose in the range 0.00–2.00 g on the removal percent of MB dye, as shown in Figure S5. It was observed that the NaCl dose did not affect the adsorption percent of MB dye in the studied range, as shown in Figures S5 and S6.

#### 3.2.9. Adsorption Mechanism

**Figure 8:**
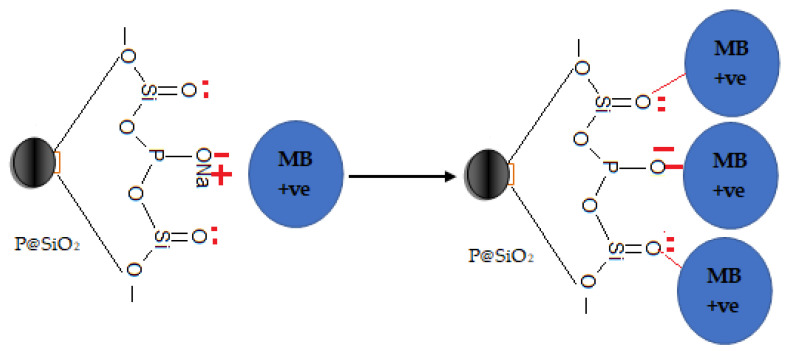
The proposed adsorption mechanism of MB dye onto P@SiO_2_ nanocomposite from aqueous media.

The P@SiO_2_ nanocomposite was prepared by combining sodium silicate and sodium phosphate. Hence, the binding sites, which were responsible for the interaction with the MB species, were mainly composed of silanol and phosphate groups. Upon contacting with the positive MB species pollutants in the aqueous media, the negative binding sites P@SiO_2_ nanocomposite interact with the MB species through ionic bond and oxygen lone pair sharing. The proposed adsorption mechanism is illustrated in Figure 8. The electrostatic attractions between the positive MB species and the negative active sites of P@SiO_2_ nanocomposite mainly depends on the reaction pH [49,50]. In an acidic environment, the H-atom will convert Si=O into Si-OH which will enhance the activity of these groups towards interaction with the positive MB species. On the other hand, as the pH of the media increases, the ionization of P-O^−^ Na^+^ groups increase, which will increase the affinity of the P@SiO_2_ nanocomposite towards the MB species. Also, the H-bonding becomes dominant in the adsorption mechanism and plays a vital role to improve the adsorption capacity [26,50]. P@SiO_2_ nanocomposite and MB dye have many N-atoms; therefore, the adsorption capacities are enhanced due to the formation of N-H…N bonds between P@SiO_2_ nanocomposite and MB species.

### 3.3. Comparison Study

A comparative evaluation of the maximum adsorption capacity of P@SiO_2_ nanocomposite to adsorb MB dye according to the Langmuir isotherm and other adsorbent materials in the literature is listed in Table 7 [49,50,51,52,53,54,55,56,57,58,59,60,61,62,63,64,65,66,67,68,69,70,71,72,73,74,75,76,77,78,79]. Referring to the recent literature, equilibrium time and adsorption capacities are the main goals for scientists to investigate and develop many novel adsorbent materials. Comparatively, the prepared P@SiO_2_ nanocomposite illustrated considerable adsorption capacity compared to many novel materials and modified activated carbon materials. On the other hand, the obtained adsorption capacity of the prepared P@SiO_2_ nanocomposite was lower than some reported adsorbents; the major advantages of the prepared adsorbent over those reported materials were the rapid adsorption rate and easy recovery from aqueous solution after the adsorption process for reuse. Also, for the prepared P@SiO_2_ nanocomposite, the equilibrium time was reached rapidly within 100 s with an adsorption capacity of 76.9 mg/g. Therefore, the prepared P@SiO_2_ nanocomposite is suggested as an available, high-potential, and promising sorbent nanocomposite to remove MB dye from an aqueous media with considerable efficiency. Also, the P@SiO_2_ nanocomposite can meet commercial needs for water treatment applications.

## 4. Conclusions and Future Perspectives

Here, we investigated a simple hydrothermal strategy to prepareP@SiO_2_ nanocomposite to efficiently remove methylene blue dye from an aqueous solution. SEM, EDX, XRD, and FTIR techniques were employed to characterize the prepared nanomaterial. Various parameters that affected the adsorption process were investigated, such as preparing P@SiO_2_ nanocomposite dose, MB dye concentration, pH, temperature, and NaCl dose in the kinetic study. An increase in the adsorbent dose leads to minimizing the amount of dye adsorbed to one gram of the adsorbent. While the adsorption capacity of MB dye (qe) increases with a further increase in the initial dye concentration. On the other hand, the maximum adsorption capacities of the MB dye have varied according to the pH values. Moreover, increasing the dye solution temperature will lead to a decrease in the maximum adsorption capacity of the adsorbate. Finally, NaCl at various doses does not affect MB adsorption. From the analysis of the experimental results, the pseudo-second-order was an excellent fit for the obtained data. Moreover, according to Langmuir isotherm, the P@SiO_2_ nanocomposite shows excellent saturation capacity (76.92 mg g^−^^1^) which was suitable compared to other adsorbents in the literature. The thermodynamic studies showed that the adsorption process is preferred at low temperatures, exothermic, and ordered at the solid/solution interface. Also, the comparison study showed the promising properties and adsorption efficiency of P@SiO_2_ nanocomposite compared with other adsorbent materials. Also, P@SiO_2_ nanocomposite can be recommended as an eco-friendly absorbent material to purify wastewater from various cationic pollutants with significant efficiency in future works.

## Figures and Tables

**Table 1:** The calculated parameters of the *pseudo*-first-order and *pseudo*-second-order kinetic model for various P@SiO_2_ nano-composite.

Adsorbent Dose, mg	q_e exp_ (mg/g)	First-Order Kinetic Parameter			Second-Order Kinetic Parameter		
		K_1_ (min^−1^)	q_e cal_ (mg/g)	R^2^	K_2_ (g mg^−1^ min^−1^)	q_e cal_ (mg/g)	R^2^
10 15 20 25	79.58 58.67 45.81 37.9	−0.31 −0.77 −0.54 −0.77	21.38 17.78 8.79 5.32	0.981 0.938 0.927 0.931	0.069 0.11 0.26 0.35	80.00 55.90 46.10 39.02	0.996 0.970 0.999 0.999

**Table 2:** The calculated parameters of the pseudo-first-order and pseudo-second-order kinetic model for various concentrations of MB dye.

[MB], ppm	q_e exp_ (mg/g)	First-Order Kinetic Parameter			Second-Order Kinetic Parameter		
		K_1_ (min^−1^)	q_e cal_ (mg/g)	R^2^	K_2_ (g mg^−1^ min^−1^)	q_e cal_ (mg/g)	R^2^
100	37.9	−0.77	5.32	0.899	0.36	38.46	0.999
150	50.85	−0.71	8.71	0.987	0.37	50.51	0.998
200	58.64	−1.14	18.88	0.988	0.24	59.17	0.998
300	73.95	−0.71	27.54	0.993	0.028	75.47	0.999

**Table 3:** The calculated parameters of the pseudo-first-order and pseudo-second-order kinetic model for various pH values.

pH	q_e exp_ (mg/g)	Pseudo-First-Order Kinetic Parameter			Pseudo-Second-Order Kinetic Parameter			
		K_1_ (min^−1^)	q_e cal_ (mg/g)	R^2^	K_2_ (g mg^−1^ min^−1^)	q_e cal_ (mg/g)	R^2^	
1.5	58.17	−1.45	9.95	0.94	0.43	58.48 ± 0.4	0.999	
3	40.27	−0.88	13.12	0.886	0.17	41.67 ± 1.1	0.999	
7	50.85	−0.71	8.17	0.927	0.36	50 ± 0.7	0.999	
9	59.80	0.00	0.00	0.000	−1.34	58.48 ± 1.5	0.999	
11	57.15	−0.37	9.55	0.64	0.16	58.14 ± 1.3	0.998	

**Table 4:** The calculated parameters of the pseudo-first-order and pseudo-second-order kinetic model at different temperatures.

T, °C	q_e exp_ (mg/g)	First-Order Kinetic Parameter			Second-Order Kinetic Parameter		
		K_1_ (min^−1^)	q_e cal_ (mg/g)	R^2^	K_2_ (g mg^−1^ min^−1^)	q_e cal_ (mg/g)	R^2^
25	59.80	0.00	0.00	0.000	−1.34	58.48 ± 0.5	0.999
45	48.41	−0.97	12.88	0.777	0.22	50 ± 0.8	0.999
60	44.74	−1.57	8.51	0.970	0.60	45.45 ± 0.3	0.999
80	42.51	−2.42	14.13	0.910	0.35	43.48 ± 0.6	0.999

**Table 5:** Calculated equilibrium constants for adsorption of MB.

Adsorbent	Langmuir Isotherm Model			Freundlich Isotherm Model			Tempkin Isotherm Model		
	Q° (mg/g)	b (L/mg)	R^2^	n	K_f_ (mg/g)	R^2^	B (J mol^−1)^	A (L mg^−1^)	R^2^
MB	76.92 ± 0.2	0.092	0.980	4.76	4.14	0.980	4.70	11.40	0.94

**Table 6:** Thermodynamic parameters for MB dye removal from aqueous media by P@SiO_2_ nanocomposite.

	T (K)	MB
−ΔG (KJ mol^−1^)	298	1.982 ± 0.09
	318	1.356 ± 0.07
	333	0.435 ± 0.005
	353	−0.088 ± 0.001
−ΔH (KJ mol^−1^)	-	13.56 ± 0.3
−ΔS (KJ mol^−1^)		0.04 ± 0.008

**Table 7:** Comparison of the maximum adsorption capacities of P@SiO_2_ with recently reported SiO_2_-based materials to remove MB dye [49,50,51,52,53,54,55,56,57,58,59,60,61,62,63,64,65,66,67,68,69,70,71,72,73,74,75,76,77,78,79].

Adsorbent	T, min	Adsorbent dose	T, °C	pH	q_e_, mg/g	Ref.
MOFs, MIL-101(Cr)	24 h	2.5 mg/10 mL	25	--	34.3	[49]
Fe_3_O_4_@SiO_2_-CR	10	30 mg/30 mL	25	11	31.44	[50]
Mesoporous Fe_3_O_4_@SiO_2_	5	1 mg/L	25	7	33.12	[51]
PLA-PEG/MgSiO_3_ Membrane	--	3	25	10	79% [MB] = 5.5 ppm	[52]
Fe_3_O_4_@SiO_2_-EDA-COOH	60	20 mg/50 mL	25	10	43.15	[53]
Monodispersed MSNs	6	5 mg/26 mL	25	7	34.23	[54]
(FA-DMSN)	6	10 mg/15 mL	25	7	90.7	[55]
CMMSNs	300	0.02 g/50 mL	25	7	43.03	[56]
SNFs-LMw	360	5 mg/10 mL	25	10	278.8	[57]
SNFs-HMw	240				123.3	
MSM@PDA	15	0.37 g	25	10	83.8	[58]
γ-Fe_2_O_3_/SiO_2_	240	2 g/L	25	7	116.09	[59]
Fe_3_O_4_@Void@m.SiO_2_	3	5 mg/15 mL	25	9	163.93	[60]
Fe_3_O_4_-graphene@mesoporous SiO_2_ Nanocomposites	15	10	40	11	0.98–102.2	[61]
Silica Xerogel Synthesized from Volcanic Tuff	60	0.0016 g/mL	40	5	51.967	[62]
Silica gel derived from Algerian siliceous	240	1 g/L	25	6.3	80.45	[63]
Cysteine-Functionalized Mesoporous Silica ((MSN-Cys)	80	10 mg/10 mL	25	8.5	140	[64]
(MSN) (MSN-NH_2_)	30	0.05 g/25 mL	--	11	2.899 1.736	[65]
βCD-SNHS	720	0.01 g/7 mL	27	10.5	60.55	[66]
Modified Nano-silica with Bismuth and Iron	20	8 g/L	25	5-6	9.54	[67]
Mesoporous Silicalite-1	240	0.10 wt%/50 mL	30		19.04	[68]
Silica Nanoparticles (SNPs)	60	0.3 g/L	30	7	31	[69]
Mesoporous Silica Spheres	20	15 mg/15 mL	30	5-7	60	[70]
AC-MnFe_2_O_4_	15	24 mg/L	30	4	77.74	[71]
Activated Charcoal from Ficus carica bast	90	0.5 g/100 mL	30	8	47.62	[72]
Activated Carbon	120	0.1 g/100 mL	30	8	72	[73]
AC1	45	2 g/L	25	9	28.65	[74]
AC2	120				17.57	
AC3	120				0.809	
BCC	60	0.1 g/20 mL	25	7	11.63	[75]
BCAC-10					12.71	
BCAC-20					16.85	
DMWTAC	30	200	20	8	53	[76]
CNZL Activated Carbon	80	100	50	--	14.493	[77]
GFSF	360	3 g/L	27	8	19.18	[78]
Carbon Nanoparticles (TPCNPs)	90	50 mg/120 mL	20	5	98	[79]
P@Si	100 s	25 mg/10 mL	25	7	76.92	This work

## Data Availability

Data is contained within the article.

## Supplementary Materials

The following supporting information can be downloaded at: https://www.mdpi.com/article/10.3390/ma16020514/s1. Figure S1: SEM images of as prepared Si-P nanoparticles; Figure S2: The relation between q_e_ vs. C_e_ at different (a) P@SiO_2_ nano-composite doses and (b) initial MB concentrations; Figure S3: The color of MB-dye variation as a function of at time 5–420 s at different pH values; Figure S4: Thermodynamic plots for MB-dye removal from aqueous media by P@SiO_2_ nano-composite; Figure S5: Effect of NaCl doses on removal percent of MB-dye onto P@SiO_2_ nanocomposite. ([P@SiO_2_] = 10–25 mg/10 mL, [MB] = 100 ppm, pH =7, T = 25 °C); Figure S6: Color variation as a function of NaCl doses at intervals 0–420 s. References [80,81] are cited in Supplementary file.
