# Supplementary material for: A Novel P@SiO2 Nano-Composite as Effective Adsorbent to Remove Methylene Blue Dye from Aqueous Media
Source: Materials (Basel). 2023 Jan 5;16(2):514. doi: 10.3390/ma16020514 (PMC9864475; doi:10.3390/ma16020514)
Supplement: Supplementary file 1 [file materials-16-00514-s001.zip › materials-2105037-Supplementary Materials.pdf]

A novel P@SiO_2 Nano-composite as Effective Adsorbent to Remove Methylene Blue Dye from Aqueous Media

A.A.Nayl[1,*], A. I. Abd-Elhamid[2], Wael A. A. Arafa[1], Ismail M. Ahmed[1], Aref M. E. AbdEl-Rahman[2], Hesham M. A. Soliman[2], Mohamed A. Abdelgawad [3], Hazim M.Ali[1], Ashraf A. Aly [4], Stefan Bräse [5,6,*]

[1] Department of Chemistry, College of Science, Jouf University, Sakaka, Aljouf 72341, Saudi Arabia.
[2] Composites and Nanostructured Materials Research Department, Advanced Technology and New Ma-terials Research Institute, City of Scientific Research and Technological Applications (SRTA-City), New Borg Al-Arab, Alexandria 21934, Egypt; ahm_ch_ibr@yahoo.com.
[3] Department of Pharmaceutical Chemistry, College of Pharmacy, Jouf University, Sakaka, Al Jouf 72341, Saudi Arabia; mhmdgwd@ju.edu.sa.
[4] Chemistry Department, Faculty of Science, Organic Division, Minia University, El-Minia 61519, Egypt; ashrafaly63@yahoo.com
[5] Institute of Organic Chemistry (IOC), Karlsruhe Institute of Technology (KIT), Fritz-Haber-Weg 6, 76133 Karlsruhe, Germany
[6]Institute of Biological and Chemical Systems – Functional Molecular Systems (IBCS-FMS), Director Hermann-von-Helmholtz-Platz 1, D-76344 Eggenstein-Leopoldshafen, Germany

* Correspondence: aanayel@ju.edu.sa or aanayl@yahoo.com (A.A.N.); stefan.braese@kit.edu (S.B.)

S1. Experimental

S1.1. Adsorption Kinetics

Pseudo first order kinetic is given by ***Lagergren equation*** [S1] Eq.1:

$$\log(q_e - q_t) = \log q_e + \frac{K_{ads}\, t}{2.303} \quad (1)$$

q_t is the amount of solute sorbed per mass of sorbent (mg/g) at any time, q_e is the amount of sorption at equilibrium time and K_{ads} (min^{-1}) is the rate constant of pseudo first order sorption.

$$q_e = \frac{(C_o - C_e)v}{1000w} \quad (2)$$

v is the volume of dye solution (mL) and w is the dry weight of the adsorbent (g)

$$q_t = \frac{(C_o - C_t)v}{1000w} \quad (3)$$

where C_t is the concentration of the dye (mg/L) at different time intervals.

A pseudo second order kinetic model is explained by ***Ho*** equation [S2], Eq. 4.

$$\frac{t}{q_t} = \frac{1}{K_2 q_e^2} + \frac{t}{q_e} \quad (4)$$

where K_2 is the pseudo second order rate constant (g $mg^{-1}min^{-1}$).

S1.2. Isotherm models

The adsorption isotherm model was required to ascribe the interaction of the adsorbed species in the liquid phase and the solid phase. Langmuir and Freundlich models were the two famous models which applied. The resulted parameters of the previous models possess valuable information about how the adsorption process occur, nature and affinity of the adsorbent.

Langmuir isotherm suggested adsorbent surface with equally energetic adsorbing sites. Its mathematical linear form is written as in Eq. 5:

$$\frac{C_e}{q_e} = \left[\frac{1}{Q^o b}\right] + \left[\frac{1}{Q^o}\right] C_e \quad (5)$$

Where q_e is the amount of solute sorbed per unit weight of adsorbent (mg/g), C_e is the equilibrium concentration of the solute in the bulk of solution (mg/L), Q^o is the monolayer adsorption capacity (mg/g) and b is a constant related to the free energy of adsorption. A plot of C_e/q_e versus C_e gives a straight line with Q^o and b determined from the slope and the intercept, respectively.

Freundlich isotherm is an empirical equation based on an exponential distribution of adsorption sites and energies. It is represented as in Eq. 6:

$$\ln q_e = \ln k_f + \frac{1}{n} \ln C_e \qquad (6)$$

Where k_f (mg/g) and n are Freundlich constants related to adsorption capacity and adsorption intensity, respectively. A linear plot of ln q_e versus ln C_e confirms the validity of the Freundlich model.

Temkin model isotherm contains a factor that explicitly taking into account of adsorbent–adsorbate interactions. The linear equation for the model is given as

$$q_e = B \ln A + B \ln C_e \qquad (7)$$

where A is the binding constant corresponding to binding energy in L mg^{-1} and B is the heat of adsorption in J mol^{-1}.

S1.3. Thermodynamic model

For the calculation of thermodynamic parameters, the following equation was used:

$$K_c = C_{ad}/C_e \qquad (8)$$

$$\Delta G^\circ = - RT \ln K_c \qquad (9)$$

$$Ln\, K_c = \frac{\Delta S^o}{R} - \frac{\Delta H^o}{RT} \qquad (10)$$

Where K_c is the equilibrium constant, C_{ad} is the amount adsorbed on the adsorbent (mg/L) at equilibrium and C_e is the equilibrium concentration in solution (mg/L). ΔG°, ΔH° and ΔS° are changes in Gibbs free energy (kJ/mol), enthalpy change

(kJ/mol) and entropy change (J.mol/K), respectively. *R* is the gas concentration (8.314 J/mol/K) and *T* is the absolute temperature (K).

S2. Results and discussion

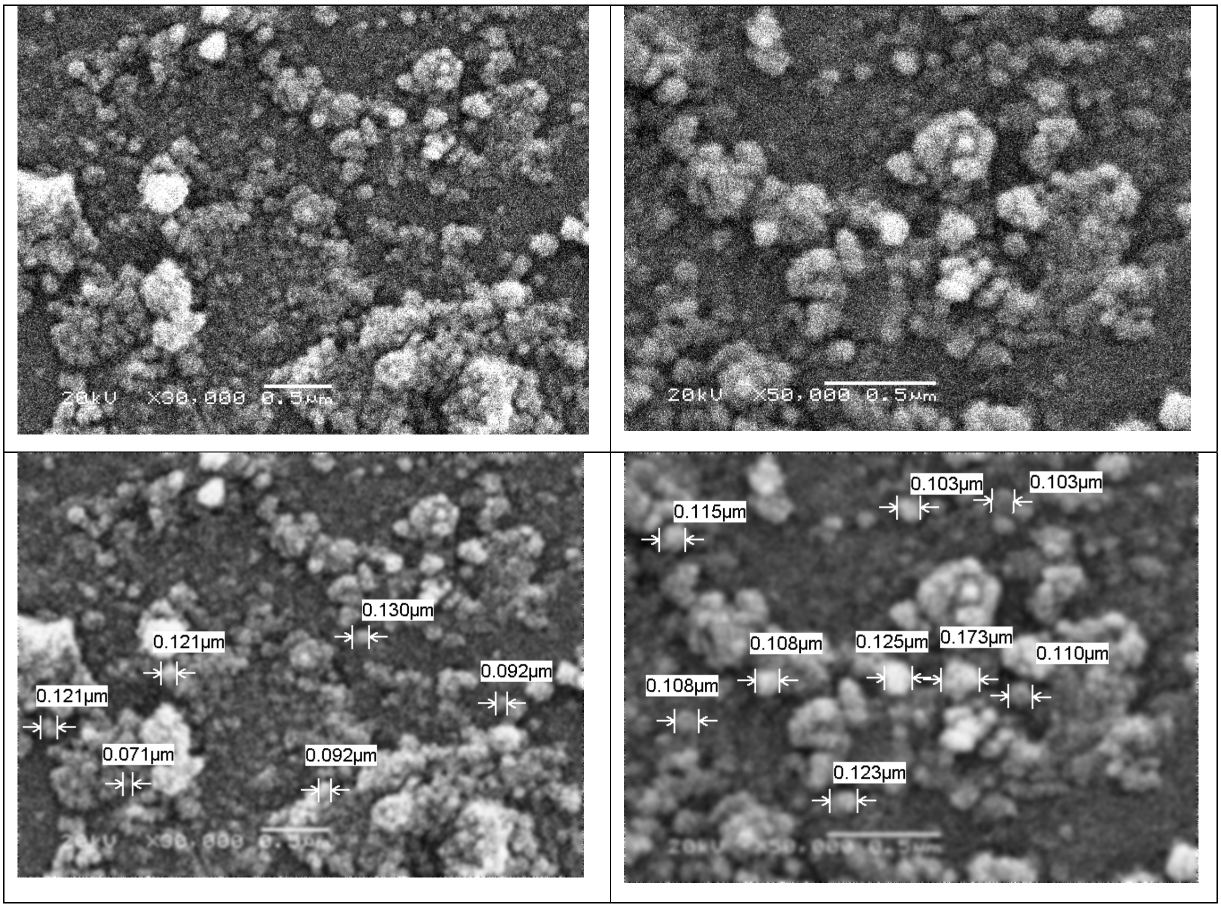

Figure S1. SEM images of as prepared Si-P nanoparticles

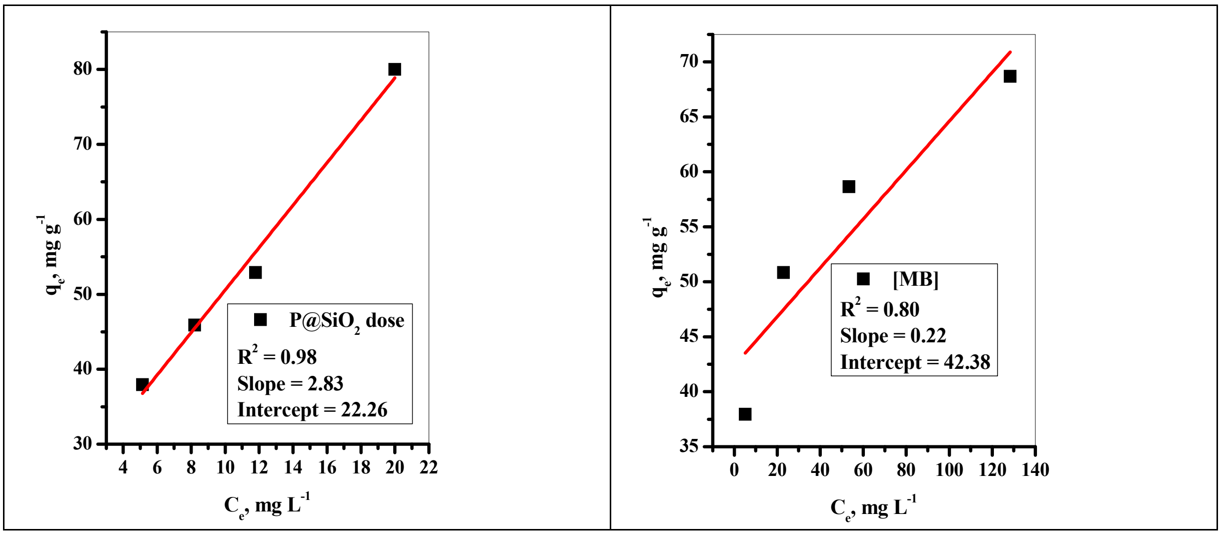

Figure S2. *The relation between* q_e vs C_e at different *a) $P@SiO_2$ nano-composite doses and b) initial MB concentrations.*
($P@SiO_2$ dose ([MB] = 100 mg/L / 10 mL, pH = 7, T = 25 °C) and b) initial MB concentrations ([$P@SiO_2$] = 25 mg/10 mL, pH = 7, T = 25 °C).

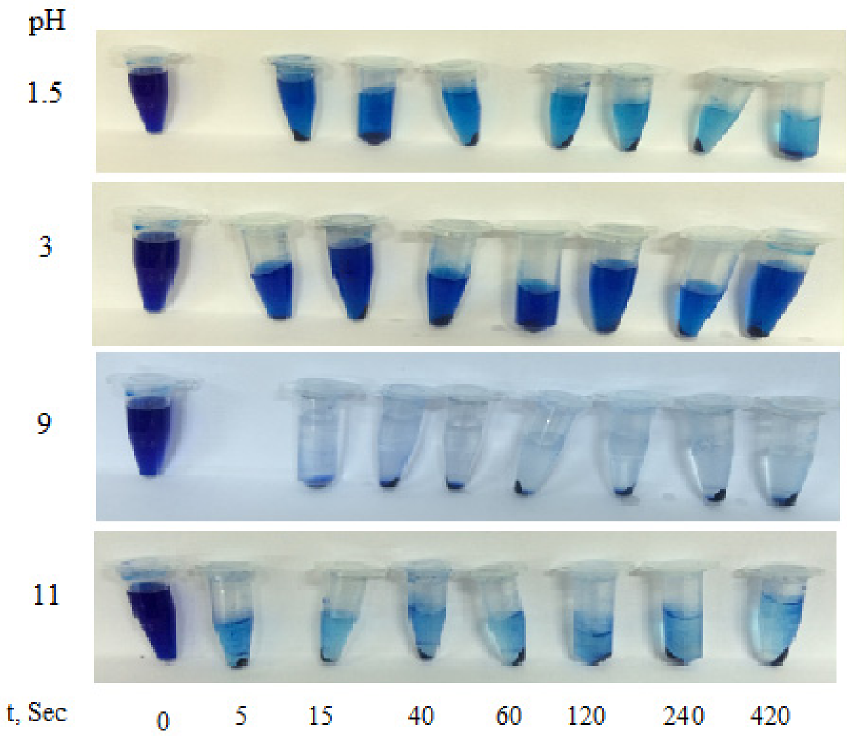

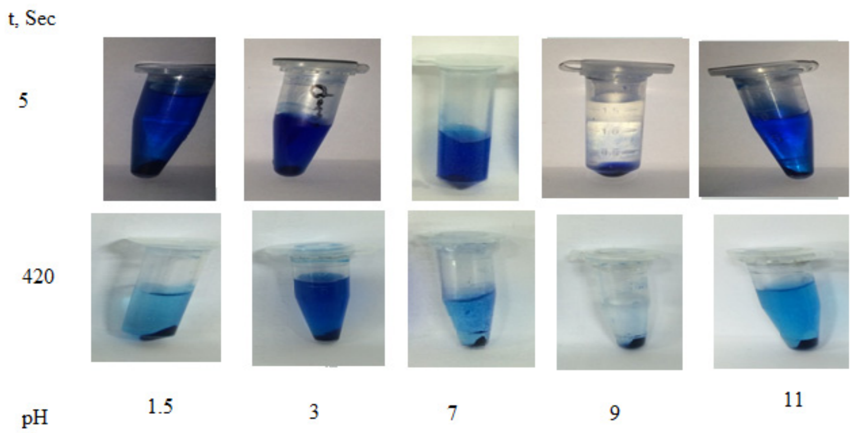

Figure S3. The color of MB-dye variation as a function of at time 5- 420 sec at different pH values.

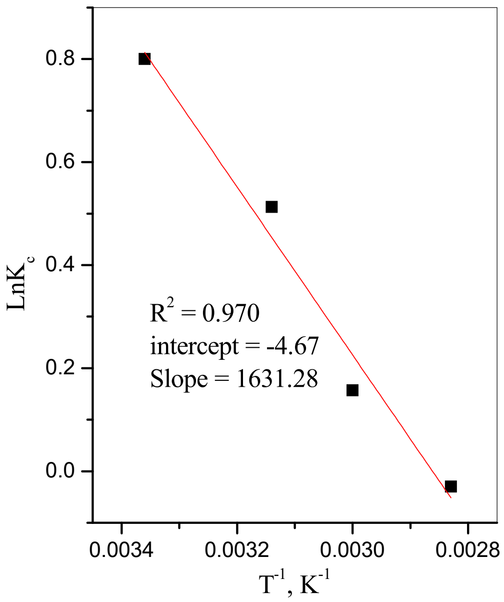

Figure S4. Thermodynamic plots for MB-dye removal from aqueous media by P@SiO_2 nano-composite.

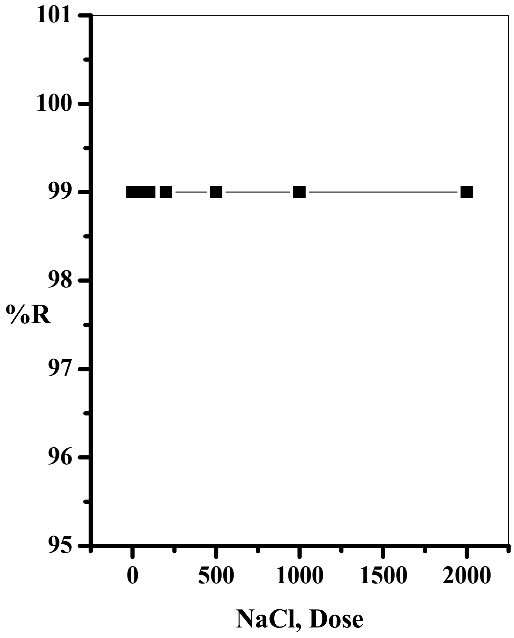

Figure S5. *Effect of NaCl doses on removal percent of MB-dye onto P@SiO_2 nanocomposite.*
([P@SiO_2] = 10-25 mg/10 mL, [MB] = 100 ppm, pH =7, T = 25 °C)

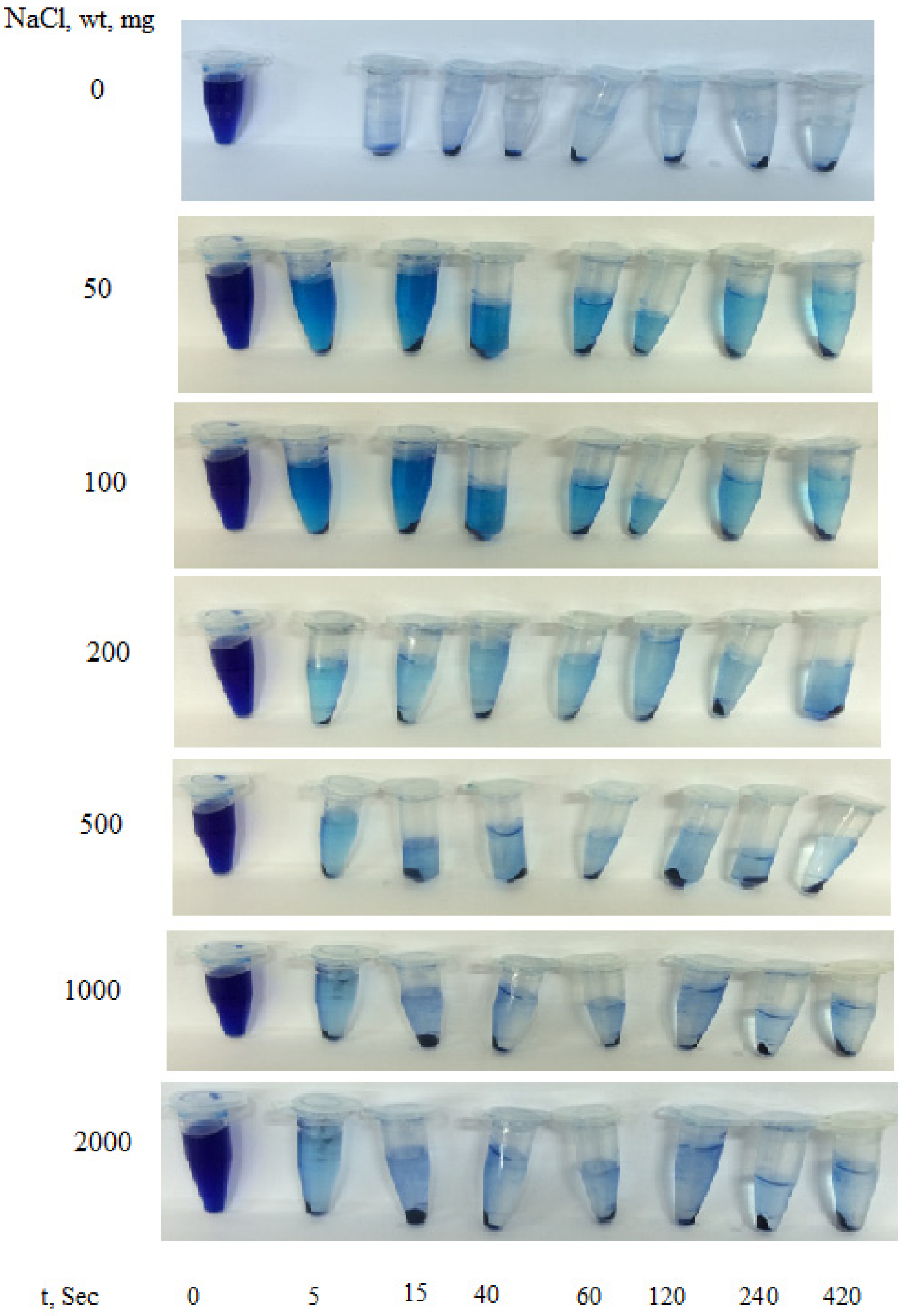

Figure S6. Color variation as a function of NaCl doses at intervals 0-420 sec.

References

S1. Lagergren, S. About the Theory of So-Called Adsorption of Soluble Substances. Kungliga Svenska Vetenskapsakademiens 526 Handlingar 1898, 24, 1 – 39. 527

S2. Ho, Y.S.; McKay, G. The sorption of lead (II) ions on peat. Water Res.1999, 33, 578 – 584. https://doi.org/10.1016/S0043-528 1354(98)00207-3.
